# Chokeberry Products and By-Products as the Potential Pharmaceuticals for Kidney Protection—An Experimental Study in Rats

**DOI:** 10.3390/plants13223136

**Published:** 2024-11-07

**Authors:** Milica Milutinović, Nada Ćujić Nikolić, Tatjana Cvetković, Katarina Šavikin, Ivana Djordjević, Ljubinka Janković Veličković, Milica Randjelović, Bojana Miladinović, Suzana Branković, Dušanka Kitić

**Affiliations:** 1 Department of Pharmacy, Faculty of Medicine, University of Niš, Bul. Dr Zorana Đinđića 81, 18000 Niš, Serbia; milica.randjelovic@medfak.ni.ac.rs (M.R.); bojana.miladinovic@medfak.ni.ac.rs (B.M.); 2 Institute of Medicinal Plants Research, Dr Josif Pančić, Tadeuša Koščuška 1, 11000 Beograd, Serbia; ncujic@mocbilja.rs (N.Ć.N.); katarina.savikin@gmail.com (K.Š.); 3 Institute of Biochemistry, Faculty of Medicine, University of Niš, Bul. Dr Zorana Đinđića 81, 18000 Niš, Serbia; tatjana.cvetkovic@medfak.ni.ac.rs; 4 Centre of Medical and Clinical Biochemistry, University Clinical Centre, Bul. Dr Zorana Đinđića 48, 18000 Niš, Serbia; 5 Pathology and Pathological Anatomy Center, University Clinical Centre, Bul. Dr Zorana Đinđića 48, 18000 Niš, Serbia; ivanadjordjevic01.12@gmail.com (I.D.); ljubinkavelickovic60@gmail.com (L.J.V.); 6 Department of Physiology, Faculty of Medicine, University of Niš, Bul. Dr Zorana Đinđića 81, 18000 Niš, Serbia; brankovic.suzana@yahoo.com

**Keywords:** chokeberry fruit, extract, waste by-products, juice, cisplatin-induced nephrotoxicity, rats, oxidative stress markers, inflammatory markers, histopathological examination

## Abstract

The study aimed to investigate the protective effects of chokeberry fruit products and by-products against cisplatin-induced acute nephrotoxicity in rats. Potential mechanisms involving oxidative stress and inflammatory responses were examined through biochemical and histopathological analyses of kidney tissue. Chokeberry waste, along with the whole fruit extract and juice, was evaluated as a potential raw material for pharmaceutical use. The chemical composition of chokeberry juice and extracts was analyzed using spectrophotometry and HPLC. Rats were treated with chokeberry preparations via intragastric tube for ten days, with a single intraperitoneal dose of cisplatin (8 mg/kg BW) administered on the third day. Post-sacrifice, plasma samples were analyzed for biochemical nephrotoxicity markers, oxidative stress, and inflammatory markers. Kidneys were removed for histopathological and biochemical analysis. Cisplatin-induced acute nephrotoxicity was confirmed by elevated plasma creatinine and blood urea nitrogen levels. Additionally, lipid peroxidation was significantly elevated, while reduced glutathione and catalase activity were significantly reduced. Pro-inflammatory mediators IL-1*β*, TNF-*α*, and IL-6 levels were significantly increased in the cisplatin group. Treatment with chokeberry extracts and juice significantly mitigated these nephrotoxic effects, as confirmed by histopathological examination and biochemical marker analysis. Notably, the waste extract demonstrated greater efficacy than the whole fruit extract, likely due to its higher concentration of polyphenolic compounds, especially anthocyanins. These results highlight the potential of chokeberry as a therapeutic and preventive agent for kidney protection, emphasizing the value of by-products rich in biologically active compounds.

## 1. Introduction

Cisplatin, also known as cis-diamminedichloroplatinum (II) or CDDP, stands as a widely employed chemotherapeutic agent in clinical oncology for the treatment of various cancer types, notably those affecting the head and neck, cervix, ovaries, and testes [1,2,3]. Its principal mode of action is the induction of DNA damage, yet its cellular effects extend beyond nuclear activities, encompassing the inhibition of DNA damage-induced transcription and apoptotic signaling. Furthermore, cisplatin induces damage to mitochondrial DNA, resulting in an elevated production of reactive oxygen species (ROS) in tissues, contributing to its cytotoxicity [3,4].

Despite its approval over four decades ago, the clinical utility of cisplatin is hampered by enduring challenges, primarily associated with its toxicity and substantial adverse effects. Thus, it is imperative to devise strategies to mitigate or eliminate these toxicities to enhance therapeutic efficacy. Acute kidney injury (AKI) represents one of cisplatin administration’s most prevalent side effects. Research indicates that cisplatin-induced renal toxicity arises from multiple factors, including the accumulation of reactive oxygen species (ROS) and oxidative stress, alongside other key mechanisms such as the activation of p53, apoptotic responses, inflammatory reactions, necrosis of renal cells and tubules, vascular dysfunction, and immune responses. The oxidative stress resulting from cisplatin-induced nephrotoxicity is linked to a reduction in endogenous antioxidant levels and the activity of antioxidant enzymes, leading to ROS accumulation within cells [1,4,5].

Recent research underscores the potential of natural antioxidants as effective agents in attenuating cisplatin-induced kidney toxicity. These antioxidants have demonstrated the ability to eliminate generated free radicals in tissues and cells by promoting the activity of endogenous enzymatic and non-enzymatic antioxidants. Additionally, they play a pivotal role in restoring the antioxidant enzymes’ capacity and protecting the mitochondria from damage, thus increasing cellular resistance to ROS [6,7,8].

Studies have confirmed that using antioxidative agents during, before, and after cisplatin therapy could improve and modulate its pharmacokinetic characteristics and enhance renal filtration [9]. This multifaceted approach may offer a promising means to decrease the toxic effects of cisplatin while preserving its therapeutic efficacy. Further investigation into applying natural antioxidants as prophylactic agents against cisplatin-induced renal damage is warranted, as it can potentially improve the overall safety and outcomes of cisplatin-based chemotherapy [10,11].

The berries of *Aronia melanocarpa*, commonly known as chokeberries, have garnered significant attention in human health due to their high content of diverse bioactive components, primarily polyphenolic compounds, vitamins, and minerals [12,13]. These berries are renowned for their exceptional antioxidant properties, making them a noteworthy natural source of potent antioxidants. Chokeberries have a historical legacy in human nutrition, tracing back to ancient times, and contemporary research categorizes them as functional foods. Recent scientific investigations have underscored the importance of chokeberry consumption in enhancing human health, substantiating their potential biological activity [12,14,15].

Plant waste, composed of discarded plant parts like stems, leaves, and peels, has emerged as a valuable resource for pharmaceutical applications. These materials contain bioactive compounds with therapeutic properties, including antioxidants and anti-inflammatory agents. Pharmaceutical research now focuses on efficient extraction methods to obtain pharmaceutical ingredients from plant waste. This sustainable approach aligns with eco-friendly practices and traditional medicine, providing a bridge between traditional and modern healthcare. Plant waste-derived pharmaceuticals also find applications in cosmetics and nutraceuticals. Innovative formulations and delivery methods enhance their effectiveness. While regulatory considerations are crucial, the future holds promise for the growth of plant waste-derived pharmaceuticals, as they meet the demand for sustainable and natural alternatives to synthetic drugs [16,17,18].

Moreover, it is worth noting that the utilization of chokeberry plant waste has also gained attention as a sustainable approach to extracting additional value from these berries. Repurposing plant waste from chokeberry processing, such as stems, leaves, and peels, can harness valuable bioactive compounds for various applications [12,18,19,20].

Therefore, our research aimed to investigate the potential protective effects of chokeberry fruit products and by-products (extract, juice, and waste extract) against cisplatin-induced acute nephrotoxicity in rats by examining possible mechanisms involving oxidative stress and the resulting inflammatory response, through biochemical and histopathological analyses of kidney tissue. It should be noted that for the first time chokeberry waste, in addition, to the whole fruit extract and juice, was tested as a potential raw material for the production of pharmaceutical preparations that could exhibit protective effects in cisplatin-induced toxicity.

## 2. Results

### 2.1. Chemical Composition of Lyophilized Chokeberry Extracts and Juice

The chemical composition of chokeberry juice and extracts was analyzed using spectrophotometry and HPLC, with results summarized in Table 1. Total polyphenols, flavonoids, anthocyanins, proanthocyanidins, and individual compounds were quantified, highlighting chokeberry preparations as abundant sources of these bio-compounds from the polyphenolic group. Notably, LCWE exhibited the highest content across nearly all quantified compounds. In comparison with LCE, extracted from whole dried berries, significantly higher amounts were revealed. LCE content of total polyphenols was almost 2 times lower than LCWE (*p* < 0.05) and 3.5 times lower when it comes to total anthocyanin (*p* < 0.05). LCJ exhibited high levels of both total and individual compounds, with total flavonoid content notably higher in the extract compared to the waste extract and juice (*p* < 0.05).

In terms of individual compound content, anthocyanins predominated over flavonoids, with cyanidin-3-*O*-galactoside being the principal compound. The whole dried berries extract (LCE) demonstrated the highest individual flavonoid content, and the highest content of individual anthocyanins was found in LCWE, as expected.

### 2.2. Effect of Chokeberry Extract, Waste Extract, and Juice on Biochemical Nephrotoxicity Markers

**Figure 1:**
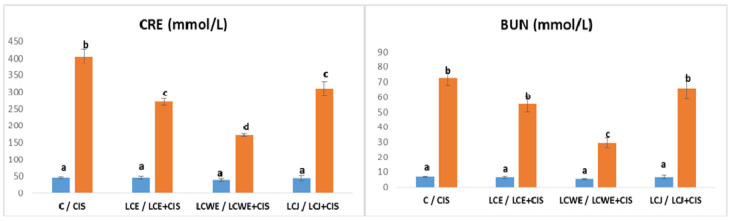
The creatinine (CRE) and blood urea nitrogen (BUN) concentrations in rat plasma. Values represent the mean ± standard deviation (*n* = 8). The bars indicate the mean values and the vertical lines represent the standard deviations. Different letters above the bars indicate a statistically significant difference in the parameter concentration between groups (*p* < 0.001; Tukey test). **C**: a control group of healthy rats administered physiological saline; **CIS**: the control group of diseased rats administered a single intraperitoneal dose of cisplatin (8 mg/kg BW); **LCE, LCWE, and LCJ**: groups of rats orally administered chokeberry extract (LCE) and waste extract (LCWE) (100 mg/kg BW) and chokeberry juice (LCJ) (1.7 mL/kg BW) for 10 days; **LCE + CIS, LCWE + CIS,** and **LCJ + CIS**: groups of rats administered LCE and LCWE (100 mg/kg BW) and LCJ (1.7 mL/kg BW for 10 days, with a single intraperitoneal dose of cisplatin (8 mg/kg BW) on the third day of the experiment. The columns represent mean values, and the vertical lines indicate standard deviations. Different letters above the bars denote statistically significant differences between groups in the concentration of biochemical parameters (*p* < 0.001, Tukey test).

Plasma concentrations of creatinine (CRE) and urea (BUN) indicated acute kidney injury, showing a significant increase in rat groups receiving intraperitoneal cisplatin (CIS group), reflecting kidney damage due to the chemotherapy agent (*p* < 0.001 compared to healthy controls). In contrast, plasma concentrations of CRE and BUN in rats administered only chokeberry preparations were similar to those in the control group, indicating no kidney damage (Figure 1).

In rats receiving cisplatin with chokeberry extracts or juice, CRE concentration was significantly elevated (*p* < 0.001) but considerably lower than in the CIS group (405.87 ± 21.18 µmol/L) (*p* < 0.001). The waste extract (LCWE) demonstrated superior protective effects compared to the fruit extract (LCE) and juice (LCJ), with the LCWE + CIS group showing the lowest CRE increase (172.12 ± 4.12 µmol/L) compared to healthy rats (45.12 ± 1.96 µmol/L). The average CRE concentration in the LCE + CIS group was 270.71 ± 9.23 µmol/L, which did not significantly differ from the LCJ + CIS group (310.12 ± 20.77 µmol/L).

Regarding plasma urea concentration (BUN), chokeberry preparations reduced BUN levels in rats with cisplatin-induced damage, with significant reduction only observed in the LCWE group (*p* < 0.001). The mean BUN concentration in the CIS group was 72.85 ± 4.92 mmol/L, significantly higher than in healthy animals (6.93 ± 0.42 mmol/L). In the LCWE + CIS group, BUN values were significantly lower (29.56 ± 3.15 mmol/L) than in the CIS group but still elevated compared to healthy controls (*p* < 0.001).

### 2.3. Effect of Chokeberry Extract, Waste Extract, and Juice on Oxidative Stress Markers

**Figure 2:**
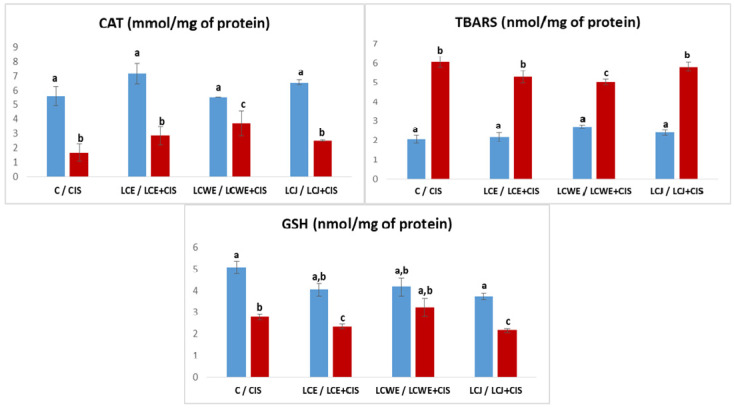
Levels of the oxidative stress markers in rat kidney tissue. Values represent the mean ± standard deviation (n = 8). The bars indicate the mean values and the vertical lines represent the standard deviations. Different letters above the bars indicate a statistically significant difference in the parameter concentration between groups (*p* < 0.001; Tukey test). **C**: a control group of healthy rats administered physiological saline; **CIS**: the control group of diseased rats administered a single intraperitoneal dose of cisplatin (8 mg/kg BW); **LCE, LCWE, and LCJ**: groups of rats orally administered chokeberry extract (LCE) and waste extract (LCWE) (100 mg/kg BW) and chokeberry juice (LCJ) (1.7 mL/kg BW) for 10 days; **LCE + CIS, LCWE + CIS,** and **LCJ + CIS**: groups of rats administered LCE and LCWE (100 mg/kg BW) and LCJ (1.7 mL/kg BW for 10 days, with a single intraperitoneal dose of cisplatin (8 mg/kg BW) on the third day of the experiment. The columns represent mean values, and the vertical lines indicate standard deviations. Different letters above the bars denote statistically significant differences between groups in the concentration of tissue oxidative stress markers (*p* < 0.001, Tukey test).

The effects of LCE, LCWE, and LCJ on the activity of catalase (CAT), the concentration of reduced glutathione (GSH), and the levels of TBARS in kidney tissue of rats with cisplatin-induced damage are shown in Figure 2.

TBARS levels indicated that cisplatin increased lipid peroxidation in kidney tissue (*p* < 0.01). Although a slight increase was also observed in rats receiving chokeberry preparations with cisplatin, TBARS values were lower than those in the CIS group. Notably, the group receiving cisplatin and chokeberry waste extract (CBWE) showed a statistically significant difference in TBARS levels compared to both healthy and diseased control groups (*p* < 0.01).

Catalase (CAT) activity, an indicator of antioxidant protection, significantly decreased due to cisplatin treatment (*p* < 0.01). While chokeberry preparations slightly increased CAT activity compared to the CIS group, levels remained significantly lower than those in the control group (*p* < 0.01). In particular, LCE treatment resulted in increased CAT activity, suggesting enhanced antioxidant protection.

The concentration of reduced glutathione (GSH), a key non-enzymatic antioxidant, significantly decreased in rats treated with cisplatin (*p* < 0.01). Chokeberry preparations did not significantly affect GSH levels, as concentrations in rats receiving LCE or LCJ with cisplatin remained significantly lower than in both healthy and diseased control groups (*p* < 0.01).

**Figure 3:**
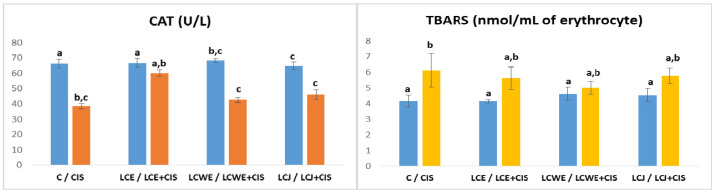
**Plasma catalase (CAT) activity and TBARS levels in rat erythrocytes.** Values represent the mean ± standard deviation (*n* = 8). The bars indicate the mean values and the vertical lines represent the standard deviations. Different letters above the bars indicate a statistically significant difference in the parameter concentration between groups (*p* < 0.001; Tukey test). **C**: a control group of healthy rats administered physiological saline; **CIS**: the control group of diseased rats administered a single intraperitoneal dose of cisplatin (8 mg/kg BW); **LCE, LCWE, and LCJ**: groups of rats orally administered chokeberry extract (LCE) and waste extract (LCWE) (100 mg/kg BW) and chokeberry juice (LCJ) (1.7 mL/kg BW) for 10 days; **LCE + CIS, LCWE + CIS,** and **LCJ + CIS**: groups of rats administered LCE and LCWE (100 mg/kg BW) and LCJ (1.7 mL/kg BW for 10 days, with a single intraperitoneal dose of cisplatin (8 mg/kg BW) on the third day of the experiment. The columns represent mean values, and the vertical lines indicate standard deviations. Different letters above the bars denote statistically significant differences between groups in the concentration of tissue oxidative stress markers (*p* < 0.001, Tukey test).

Catalase (CAT) activity was also assessed in plasma, as well as the concentration of thiobarbituric acid reactive substances (TBARS) in erythrocytes (Figure 3).

CAT activity in the plasma of rats significantly decreased due to cisplatin’s harmful effects (*p* < 0.01). However, in the LCE + CIS group, CAT activity increased significantly compared to the CIS group (*p* < 0.01), with values not differing significantly from those in the healthy control group. In contrast, plasma CAT activity in the LCWE and LCJ groups remained reduced and did not significantly mitigate the effects of the cytostatic.

TBARS levels indicated a significant increase in lipid peroxidation in erythrocytes of animals treated with cisplatin (*p* < 0.01). While the differences in TBARS levels for the protective effects of the tested preparations were not significant compared to the CIS group, there was a reduction in lipid peroxidation in erythrocytes. Notably, CBWE showed the most significant impact on reducing TBARS concentration compared to the other two preparations.

### 2.4. Effect of Chokeberry Extract, Waste Extract, and Juice on Inflammatory Markers

**Figure 4:**
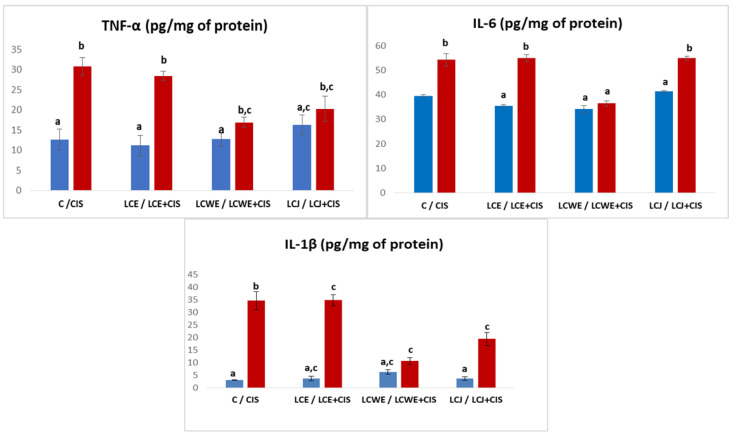
**Levels of the inflammatory markers in rat kidney tissue.** Values represent the mean ± standard deviation (*n* = 8). The bars indicate the mean values and the vertical lines represent the standard deviations. Different letters above the bars indicate a statistically significant difference in the parameter concentration between groups (*p* < 0.001; Tukey test). **C**: a control group of healthy rats administered physiological saline; **CIS**: the control group of diseased rats administered a single intraperitoneal dose of cisplatin (8 mg/kg BW); **LCE, LCWE, and LCJ**: groups of rats orally administered chokeberry extract (LCE) and waste extract (LCWE) (100 mg/kg BW) and chokeberry juice (LCJ) (1.7 mL/kg BW) for 10 days; **LCE + CIS, LCWE + CIS,** and **LCJ + CIS**: groups of rats administered LCE and LCWE (100 mg/kg BW) and LCJ (1.7 mL/kg BW for 10 days, with a single intraperitoneal dose of cisplatin (8 mg/kg BW) on the third day of the experiment. The columns represent mean values, and the vertical lines indicate standard deviations. Different letters above the bars denote statistically significant differences between groups in the concentration of tissue oxidative stress markers (*p* < 0.001, Tukey test).

The concentrations of inflammatory cytokines TNF-α, IL-6, and IL-1*β* in rat kidney tissue demonstrated a significant increase in rats treated with cisplatin (*p* < 0.01), with the highest increase seen for IL-1*β* (Figure 4). Chokeberry preparations effectively reduced inflammation caused by cisplatin-induced kidney damage, as indicated by lower levels of all measured cytokines compared to the CIS group. Notably, LCWE and LCJ showed more pronounced protective effects than LCE.

**Figure 5:**
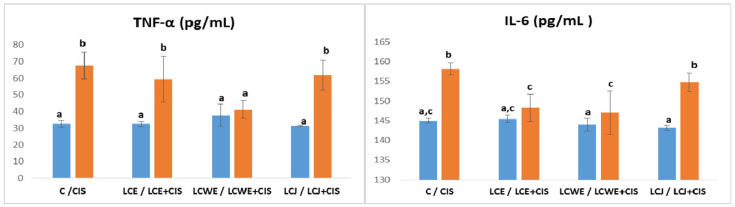
**Plasma inflammatory markers (TNF-*α* and IL-6) levels.** Values represent the mean ± standard deviation (*n* = 8). The bars indicate the mean values and the vertical lines represent the standard deviations. Different letters above the bars indicate a statistically significant difference in the parameter concentration between groups (*p* < 0.001; Tukey test). **C**: a control group of healthy rats administered physiological saline; **CIS**: the control group of diseased rats administered a single intraperitoneal dose of cisplatin (8 mg/kg BW); **LCE, LCWE, and LCJ**: groups of rats orally administered chokeberry extract (LCE) and waste extract (LCWE) (100 mg/kg BW) and chokeberry juice (LCJ) (1.7 mL/kg BW) for 10 days; **LCE + CIS, LCWE + CIS,** and **LCJ + CIS**: groups of rats administered LCE and LCWE (100 mg/kg BW) and LCJ (1.7 mL/kg BW for 10 days, with a single intraperitoneal dose of cisplatin (8 mg/kg BW) on the third day of the experiment. The columns represent mean values, and the vertical lines indicate standard deviations. Different letters above the bars denote statistically significant differences between groups in the concentration of tissue oxidative stress markers (*p* < 0.001, Tukey test).

The cytokine levels in the plasma of rats treated with cisplatin were significantly higher compared to the control group of healthy rats (*p* < 0.01). The concentration of TNF-α was increased in the groups that received aronia extract (LCE) and juice (LCJ) in combination with cisplatin compared to the control (C) (*p* < 0.01) but slightly decreased compared to the concentration in the cisplatin-treated group (CIS). A statistically significant reduction in TNF-α concentration relative to the CIS group was observed only in the LCWE + CIS group (*p* < 0.01) (Figure 5).

Aronia extracts led to a significant reduction in IL-6 levels in the groups treated with cisplatin compared to the levels in the control cisplatin-treated group (*p* < 0.01). The concentrations did not statistically differ from those in the control group (C), indicating pronounced protective effects of the extracts, particularly LCWE. Aronia juice did not show a significant reduction in cytokine levels compared to the concentration in the CIS group (Figure 5).

### 2.5. Histopathological Analyses of Kidney Tissue

**Figure 6:**
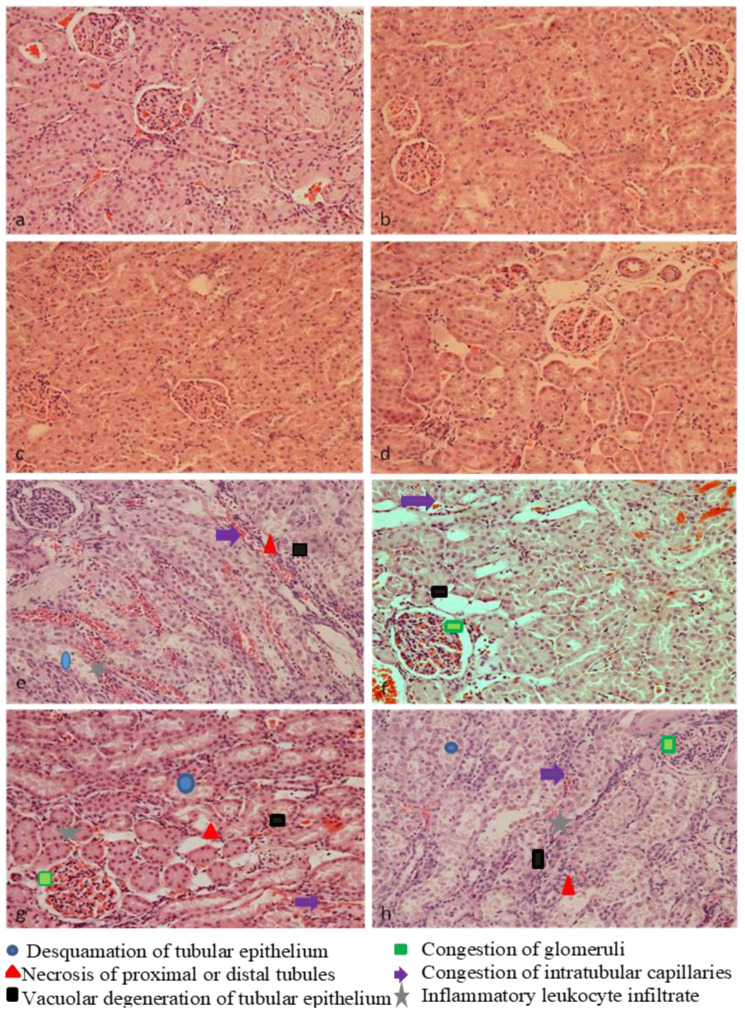
**Morphological alterations in rat kidney tissue (H&E, 200×);** (**a**)—control; (**b**)—lyophilized chokeberry extract (LCE); (**c**)—lyophilized chokeberry waste extract (LCWE); (**d**)—lyophilized chokeberry juice (LCJ); (**e**)—cisplatin; (**f**)—cisplatin + LCE; (**g**)—cisplatin + LCWE; (**h**)—cisplatin + LCJ.

**Figure 7:**
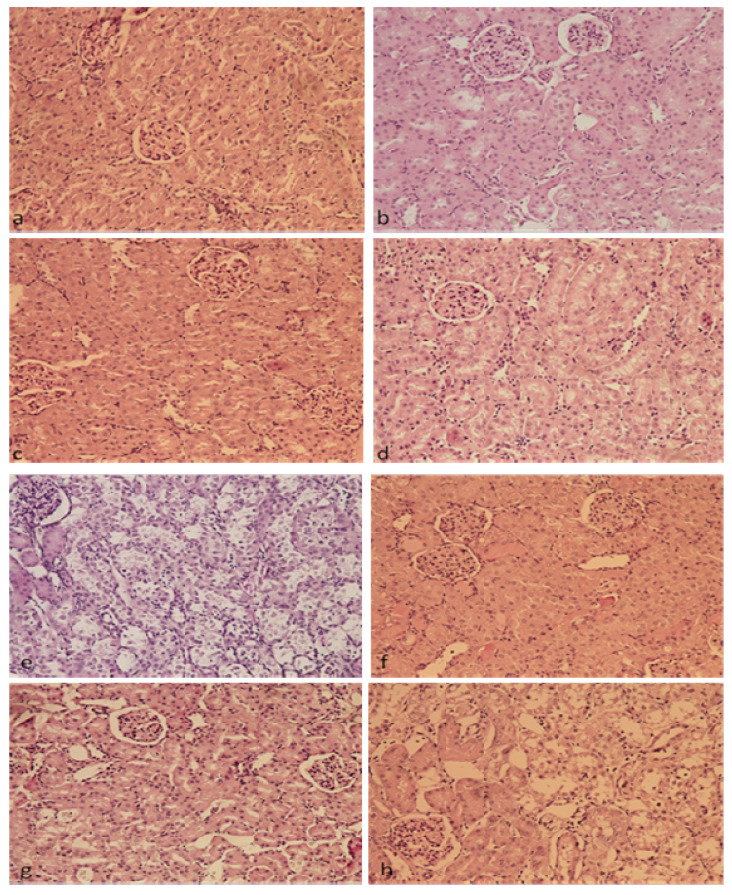
**Morphological alterations in the basal membrane and cytoplasm of tubular epithelium of the rat kidney tissue (PAS, 200×);** (**a**)—control; (**b**)—lyophilized chokeberry extract (LCE); (**c**)—lyophilized chokeberry waste extract (LCWE); (**d**)—lyophilized chokeberry juice (LCJ); (**e**)—cisplatin; (**f**)—cisplatin + LCE; (**g**)—cisplatin + LCWE; (**h**)—cisplatin + LCJ.

Table 2 shows the degree of kidney damage in different groups of rats according to a predefined scale: up to 25% (−), from 25 to 50% (+), from 50 to 75% (++), and greater than 75% (+++). Histopathological analysis of kidney tissue (Figure 6 and Figure 7) from the control group of rats (a), as well as rats treated with chokeberry extracts and juice (b–d), indicates unchanged kidney morphology, with normal-looking glomeruli and tubules. In the group of rats treated with a single dose of cisplatin at 8 mg/kg, altered kidney tissue morphology was observed, with pronounced vacuolar and fatty degeneration, as well as desquamation of the tubular epithelium and damage to the brush border of the proximal tubules. There is congestion of the intertubular capillaries with moderate inflammatory infiltrate. Focal necrosis was observed in some proximal and distal tubules. Additionally, a reduction in the diameter of some glomeruli and congestion of glomerular capillaries was noted. Periodic acid-Schiff (PAS) staining was used to detect damage to the basal membrane and cytoplasm of the tubular epithelium.

Histopathological findings of kidney tissue from rats treated with cisplatin and chokeberry preparations showed less pronounced changes compared to the group treated with the cytostatic alone, indicating the protective effects of these preparations. Table 2 shows that the most pronounced protective effect was seen with chokeberry extract (LCE), which was used to treat rats after a single dose of cisplatin (8 mg/kg BW) (Figure 6f and Figure 7f), compared to the effects of LCWE and LCJ. It was shown that chokeberry extract significantly reduced the desquamation and damage to the brush border of the proximal tubule epithelium, which was prominent in the group of rats treated only with cisplatin. The chokeberry extract also reduced the necrosis of the proximal and distal kidney tubules observed in the cisplatin-treated group, as well as vacuolar and fatty degeneration of the tubular epithelium. Congestion of glomeruli and intertubular capillaries was noticeably reduced compared to the damage caused by cisplatin (Figure 6e and Figure 7e). Inflammatory infiltrate was present in the groups treated with LCWE and LCJ in combination with the cytostatic, while it was absent in the group treated with LCE and cisplatin. In the group of rats treated with LCWE in combination with the cytostatic, there were minor improvements in terms of desquamation of the tubular epithelium, brush border damage, and congestion of intertubular capillaries (Figure 6g and Figure 7g). Chokeberry juice showed the weakest protective effect, which was evident only as a reduction in desquamation of the kidney tubule epithelium (Figure 6h and Figure 7h). The reduction in glomerular diameter observed in the cisplatin-treated group was less pronounced only in rats treated with the extract, while the other two preparations did not prevent this damage.

## 3. Discussion

The escalating prevalence of chronic non-communicable diseases, including cancer, poses a formidable public health challenge in developed nations. Consequently, extensive endeavors have been dedicated to devising effective strategies for disease prevention and management, as well as for the prevention of complications that arise due to available therapy [21]. In recent years, growing interest has centered on the potential utility of chokeberry and its derivatives in averting and treating these maladies [15]. Moreover, accumulating evidence suggests the feasibility of employing chokeberry in addressing a spectrum of other health concerns, encompassing ailments such as respiratory infections, select inflammatory conditions, and disorders of the cardiovascular and nervous systems. Therefore, consuming chokeberries and their preparations may help prevent certain chronic diseases, and could also mitigate the toxic effects of some xenobiotics on human health [15,22,23].

Numerous compounds have been studied as potential agents to prevent or mitigate cisplatin nephrotoxicity [5]. Plant extracts and their active metabolites are increasingly studied for their potential to minimize cisplatin-induced nephrotoxicity. Many studies have confirmed the in vivo effects of specific plant extracts on animal models and the protective effects of isolated compounds, primarily due to their antioxidant properties. The mechanism by which plant substances reduce cisplatin nephrotoxicity is only partially understood and is still the focus of the research [5,24,25].

In a cisplatin-induced pathological response, oxidative stress is an early reaction that causes varying degrees of damage to intracellular components. Reactive oxygen species (ROS) act as initial signaling molecules, leading to a cascade of pathological processes such as apoptosis and necrosis. The mechanism of ROS generation induced by cisplatin is strongly associated with the depletion of endogenous antioxidants, damage to antioxidant enzymes, mitochondrial crosstalk with the endoplasmic reticulum via ROS and Ca^2+^, and disruption of the cytochrome P450 (CYP) system in the endoplasmic reticulum. These processes contribute to the excessive buildup of intracellular ROS and oxidative stress. Additionally, research has shown that natural antioxidants can protect against cisplatin-induced nephrotoxicity by reducing or eliminating excess free radicals and impacting other non-redox pathways [5].

In most studies, cisplatin, as a cytostatic drug, caused kidney damage by entering kidney epithelial cells and causing DNA damage and endothelial dysfunction. This leads to apoptosis and necrosis due to ROS production and activation of mitochondrial or non-mitochondrial apoptotic pathways, triggering a strong inflammatory response [26,27].

Catechin, epigallocatechin gallate, and epigallocatechin, as polyphenolic compounds, effectively protect against nephrotoxicity [28]. Studies on mice treated with cisplatin showed that pretreatment with these polyphenols reduced toxicity and improved kidney function [29]. Proanthocyanidins can inhibit cell apoptosis and necrosis and prevent drug-induced toxicity [30]. Proanthocyanidins from grape seeds have been shown to reduce drug-induced nephrotoxicity, particularly from chemotherapeutics, by preventing oxidative stress, genomic instability, and cell death [31]. Studies also show the protective effects of flavonoid quercetin on cisplatin-induced nephrotoxicity in rats [32], by reducing oxidative stress, inflammation, and apoptosis [33].

Chemical characterization results indicate a high content of polyphenolic compounds, anthocyanins, and proanthocyanidins in chokeberry extracts and juice. Specifically, the waste extract (LCWE) had the highest levels of total polyphenols, anthocyanins, and proanthocyanidins, while the extract contained the highest amount of total flavonoids. The predominant individual anthocyanin was cyanidin-3-*O*-galactoside, as also reported by previous studies [34,35,36].

Research has shown that the content of active compounds, especially polyphenols, is directly correlated with the antioxidant activity of chokeberry preparations. Specifically, a study conducted by Milutinović et al. (2024) demonstrated that chokeberry extract, waste, and juice exhibit significant antioxidant activity in vitro [36]. In our study, the lyophilization method was used to preserve as many active principles as possible, as confirmed by the previous research [34,36]. Lyophilization, or freeze-drying, is a commonly applied drying method in the industry used to preserve thermolabile active principles and maintain their nutritional and health values. Research examining the impact of different drying methods (oven drying, spray drying, and lyophilization) on the preservation of polyphenol content in chokeberry juice showed that lyophilization resulted in the highest retention of active compounds [37].

Chokeberry extracts and juice administered orally for ten days exhibit protective effects against cisplatin-induced kidney damage. The rise in CRE and BUN in rats treated with chokeberry preparations was significantly lower compared to those treated with cisplatin alone. Among the tested preparations (LCE, LCWE, and LCJ), the LCWE showed the most pronounced protective effect regarding CRE and BUN levels. Therefore, we can assume that the protective effects of chokeberry preparations in cisplatin-induced kidney toxicity could be a result of their pronounced antioxidant activity, which is attributed to the high content of active compounds.

These results indicate the use of chokeberry waste by-products in the prevention of drug toxicity, for the first time. This can be very useful considering that this material remains largely unused and is very rich in active principles. This approach not only minimizes waste but also aligns with the principles of circular economy and sustainability. Research efforts in this direction are exploring methods to extract bioactive compounds from chokeberry waste for potential applications in food additives, nutraceuticals, or even natural colorants [18,19]. That potential could be extended to the pharmaceutical industry as well.

Histopathological analysis confirmed kidney damage with altered tissue morphology, vacuolar and fatty degeneration, desquamation of proximal tubular epithelium, and brush border damage, as well as moderate inflammatory infiltrate and intertubular capillary congestion. The glomeruli showed reduced diameter and capillary congestion with focal tubular necrosis.

Previous studies confirm cisplatin-induced kidney morphological changes, showing degeneration and necrosis of renal tubules consistent with our results [38,39]. LCE has the most pronounced protective effects on cisplatin-induced morphological changes in kidney tissue, primarily localized in the kidney tubules. Chemical analysis of the LCE showed that it contains flavonoids in higher concentrations compared to the LCWE and LCJ, which could explain its superior effects in terms of morphological changes. These findings are supported by earlier research [40,41,42].

Doma et al. (2023) found a correlation between the levels of certain oxidative stress biomarkers and the trace elements essential for enzyme activity. Their results showed that *A. melanocarpa* aqueous extract exhibits significant antioxidant activity and could reduce cisplatin-induced oxidative stress through the activity of oxidative stress markers in plasma correlated with the main trace elements levels in plasma and tissue [43]. Previous research has also linked oxidative stress to cisplatin-induced nephrotoxicity [44].

It is noteworthy that the dose of intraperitoneally administered cisplatin in the study by Doma et al. (2023) [43] was nearly double the dose we used, which we determined through a preliminary pilot experiment with fewer rats to cause the least animal mortality [45]. This pilot study helped us establish the dose, administration method of the chokeberry extracts and juice, and the experiment duration, forming the methodology for our current research. Additionally, the duration of our experiment was three times shorter, which can be attributed to the preparation of extracts using lyophilization and their intragastric administration via a tube to ensure the exact dose was ingested. In contrast, Doma et al. (2023) provided the chokeberry extract to the rats in place of their drinking water, leading to uncertainty about the exact amount each rat consumed, potentially extending the study duration. Nonetheless, their results align with ours, confirming the reduction of oxidative stress by using various chokeberry preparations [43].

Catalase (CAT) activity indicates effective hydrogen peroxide removal, protecting organs from oxidative damage [46]. CAT activity was examined in rat plasma and kidney tissue. In both cases, CAT activity significantly decreased in the cisplatin-treated group, suggesting reduced antioxidant protection. Enhanced antioxidant protection through exogenous agents with anti-inflammatory and cytoprotective properties may preserve kidney function [47].

We measured oxidative and inflammatory markers of kidney damage in both plasma and kidney tissue to obtain a comprehensive understanding of the disease pathology. Plasma markers provide information on systemic changes and are easily accessible for clinical assessments, while kidney tissue markers offer direct insights into local damage within the organ. This dual approach allows us to differentiate between systemic and local effects, validate our findings through cross-comparison, and understand the progression of kidney disease more effectively. Additionally, identifying markers elevated in both plasma and tissue can aid in the development of biomarkers for early diagnosis, monitoring disease progression, and evaluating therapeutic efficacy [48]. The levels of oxidative and inflammatory markers in plasma mirrored those in kidney tissue, confirming the occurrence of both local and systemic damage due to cisplatin toxicity.

Free radicals, by-products of oxidative metabolism, can damage cellular macromolecules like DNA, lipids, and proteins, leading to lipid peroxidation and protein modification [49]. Thiobarbituric acid reactive substances (TBARS) indicate lipid peroxidation and protein modification can inactivate enzymes like catalase [50]. Another balance indicator is the depletion of reduced glutathione (GSH) and protein thiols due to reactions with reactive oxygen species. GSH is an essential intracellular reducing agent maintaining thiol groups in proteins and antioxidants [51].

In our study, ROS accumulation in the kidneys was higher in cisplatin-exposed rats than in those protected by chokeberry preparations. Cisplatin significantly increased TBARS levels in erythrocytes, but prior treatment with chokeberry extracts and juice slightly reduced these levels. Earlier studies showed that flavonoid treatment inhibits lipid peroxidation and tissue damage by preventing free radical formation. Cisplatin significantly reduced the activity of antioxidant enzymes like catalase, the first line of defense against oxidative damage [52,53]. GSH, the second line of defense, converts toxic radicals to non-toxic products or removes free radicals. Cisplatin depletes GSH and protein thiols [54]. Our results confirmed that cisplatin significantly reduced GSH levels in kidney tissue compared to controls. Pretreatment with chokeberry preparations slightly increased GSH levels, particularly with LCWE. LCWE also showed the strongest protection against lipid peroxidation, likely due to its high concentration of active compounds.

Cytokines play crucial roles in cell physiology, immune response, inflammation, and tissue repair. In cisplatin-induced inflammation, TNF-*α* is a key regulatory cytokine. Inhibiting TNF-*α* with pharmacological agents or antibodies significantly reduces the production of other cytokines and kidney damage [55]. TNF-*α*, produced primarily by kidney cells, plays a crucial role in this inflammatory response [56]. Increased TNF-*α* levels in cisplatin-induced kidney damage are accompanied by rises in IL-8, IL-1*β*, and IL-18, further promoting inflammation and damage [57,58]. IL-6, an important anti-inflammatory cytokine, helps protect against nephrotoxicity by enhancing antioxidant enzyme regulation. Conversely, its genetic deletion increases oxidative stress in damaged kidneys [58,59].

Our study found that chokeberry preparations significantly reduced TNF-*α*, IL-6, and IL-1ß levels in rat kidney tissue, with LCWE showing the best effects. Chokeberry extracts and juice can potentially mitigate cisplatin-induced kidney damage by suppressing the inflammatory response.

Given all the above considerations, antioxidants may offer a new and promising approach to treating drug-induced nephrotoxicity [60]. Cancer therapies often come with serious side effects, largely due to oxidative stress, inflammatory processes, and cell apoptosis. Numerous studies have confirmed the beneficial role of antioxidants and anti-inflammatory compounds found in herbal medicinal preparations in mitigating cisplatin-induced nephrotoxicity [25]. However, it is recommended to use these preparations under continuous medical supervision, as there are some controversies regarding their use alongside anticancer drugs. Excessive use of antioxidants has been shown to potentially impact the effectiveness of chemotherapy and treatment [61]. In addition to its well-documented antioxidative and anti-inflammatory effects, chokeberry extract may exert nephroprotective effects through other mechanisms that warrant further exploration [62,63]. For example, mitochondrial protection could be a key factor, as mitochondrial dysfunction is central to cisplatin-induced nephrotoxicity. Chokeberry extract may help maintain mitochondrial integrity by reducing oxidative damage and preventing excessive mitochondrial ROS production, while also promoting mitochondrial biogenesis via pathways like PGC-1*α* [64,65]. Furthermore, the regulation of apoptosis is another potential mechanism, as chokeberry extract may counteract cisplatin-induced apoptosis by modulating the balance between pro-apoptotic and anti-apoptotic proteins such as Bax and Bcl-2 [66,67]. Additionally, its ability to improve endothelial function through enhanced nitric oxide (NO) production could offer vascular protection and improve blood flow in the kidneys [68,69]. Finally, chokeberry extract may influence autophagy-related pathways, enabling renal cells to clear damaged proteins and organelles more effectively, thereby reducing injury [70,71]. While these mechanisms are beyond the scope of our current study, they represent promising avenues for future research into the nephroprotective properties of chokeberry extract.

Despite the promising findings regarding the nephroprotective potential of chokeberry extracts and juice against cisplatin-induced acute kidney injury in rats, several limitations warrant consideration. Additionally, while using a rat model provides valuable insights, physiological differences between species may limit the applicability of findings to humans. Future research should address these limitations by evaluating diverse animal models and elucidating the underlying molecular mechanisms of chokeberry’s effects. Furthermore, clinical trials are essential to assess the safety and efficacy of chokeberry extract in human patients, while investigating potential combination therapies and broader applications in various kidney injury models could enhance our understanding of its therapeutic potential.

## 4. Materials and Methods

### 4.1. Plant Material, Extracts, and Juice Preparation

Chokeberry extracts and juice were prepared from organically grown plants harvested in July 2022 in western Serbia. Juice was obtained by pressing thawed berries with a Hydropress 90 L (Speidel, Oberderdingen, Germany), leaving by-product material for the “waste” extract. Both whole berries and waste material were dried at 40 °C for 48 h, ground, and sifted with a 0.75 sieve (Yugoslavian Pharmacopeia). Extraction was performed by soaking the dried material in 50% ethanol at a 1:20 solid-to-solvent ratio, following optimal maceration conditions for polyphenol extraction [34,35,36]. The extracts were processed to remove ethanol using a rotary vacuum evaporator (Buchi rotavapor R-114, Buchi, Labortechnik AG, Switzerland) at 60 °C to protect heat-sensitive polyphenols. The chokeberry juice, extract, and waste extract were then lyophilized: samples were frozen at −80 °C for 1 h, then freeze-dried (Beta 1–8 Freeze Dryer, Martin Christ, Harz, Germany) at −60 °C and 0.011 mbar for 24 h, with an additional hour at 0.0012 mbar to remove residual water. The lyophilized juice (LCJ), extract (LCE), and waste extract (LCWE) were stored in glass bottles at −4 °C until analysis.

### 4.2. Chemical Characterization of the LCE, LCWE, and LCJ

The analysis of total compounds within the polyphenol group (including total phenolics, anthocyanins, proanthocyanidins, and flavonoids) in the lyophilized samples (both extracts and juice) was conducted using methods outlined in previous research by Milutinović et al. (2020) [35]. Total polyphenol amounts were expressed in milligrams of gallic acid, total anthocyanins as milligrams of cyanidin-3-*O*-glucoside, total flavonoids as quercetin equivalents, and total proanthocyanins as milligrams of catechin equivalents per gram of lyophilized samples.

For the individual compounds (anthocyanins and flavonoids) present in the extracts and juice, high-performance liquid chromatography (HPLC) analysis was carried out using an Agilent series 1200 RR system (Agilent, Waldbronn, Germany) equipped with a diode array detector (DAD) and a Lichrospher RP-18 (Agilent) analytical column (250 × 4 mm i.d., 5 µm particle size) in reverse phase mode. The methodology for this analysis was previously documented by Ćujić et al. in 2018 [34]. Results were reported in milligrams per gram of lyophilized weight.

Both total and individual compound analyses were performed as triplicate measurements, and mean values ± standard deviation were calculated for accuracy and reliability.

### 4.3. Animals and Experimental Design

The experimental study included 64 male Wistar albino rats with an average body weight of 250 g. The animals were kept under strictly controlled conditions of the external environment, in the Vivarium of the Scientific Research Center for Biomedicine, Faculty of Medicine, University of Niš, Serbia. All experimental procedures with animals were approved by the Ethics Committee and are under the European Directive 2010/63/EU for experiments on animals (permits of the Ethics Committee of the Ministry of Agriculture, Forestry and Water Management—Veterinary Administration, decision number 323-07-01762/2019-05/1).

The rats were housed in clean, polycarbonate cages with a twelve-hour day-night cycle at a room temperature of 20 ± 2 °C. Water and commercial food specially adapted for rats were available to the animals ad libitum. After an adaptation period of seven days, all experimental animals were divided into eight groups (n = 8 per group). Cisplatin (Pfizer (Perth) PTY. Limited, Perth, Australia) 1 mg/mL infusion solution) was intraperitoneally administered to the rats in a dose of 8 mg/kg body weight (BW) on the third day of the experiment [63]. LCE, LCWE, and LCJ were orally administered (through an intragastric tube, reconstituted in water before administration) during the 10 days of the experiment, at 100 mg/kg BW for LCE and LCWE and 1.7 mL/kg BW for LCJ. Groups LCE + CIS, LCWE + CIS, and LCJ + CIS received the same doses of chokeberry preparations and cisplatin, as described. Group C (negative control) received 1 mL of saline daily, while Group CIS (positive control) received saline and cisplatin.

Before conducting the main study, a preliminary pilot experiment was performed to determine the optimal study conditions [45]. This initial phase involved a small number of experimental animals and helped refine the experimental protocols. The insights gained from the preliminary study ensured the reliability and validity of the conditions used in the subsequent larger-scale investigation. Several factors were considered during dose selection, including ensuring animal safety and well-being, minimizing stress during administration, and maintaining the solubility and stability of the preparations for consistent delivery via intragastric tube. The chosen doses allowed us to administer the preparations daily, without interfering with the animals’ regular activities or causing undue stress.

#### 4.3.1. Sample Collection

After the end of the experiment, on the 11th day, the rats were sacrificed with an overdose of anesthetic (Ketamidor 10%, Richter Pharma AG, Wels, Austria). Following sacrifice, 2 mL of blood was drawn from the aorta for biochemical analyses, and plasma was separated by centrifugation at 4000 rpm for 20 min. The kidneys were removed, rinsed with ice-cold saline, and divided for histopathological examination (fixed in 10% formalin) and biochemical analysis. For biochemical analysis, kidney tissue was minced, homogenized in ice-cold deionized water to create 10% homogenates using a tissue homogenizer (Xenox, Carl Roth GmbH, Karlsruhe, Germany), and centrifuged at 1500× *g* for 10 min at 4 °C. The homogenates were then stored in sterile Eppendorf tubes at −80 °C until analysis.

#### 4.3.2. Determination of Biochemical Nephrotoxicity Markers

Biochemical analyses in plasma samples were performed in the laboratory of the Clinic for Nephrology and Hemodialysis of the Univesity Clinical Center in Niš using an automatic analyzer ERBA XL-600 (ERBA Diagnostics Mannheim GmbH, Mannheim, Germany). Plasma concentrations of creatinine (CRE) and blood urea nitrogen (BUN) were determined as indicators of acute kidney injury.

#### 4.3.3. Determination of Tissue Protein Concentration

Protein concentration in kidney tissue homogenates was determined by the Lowry method using bovine serum albumin as a standard [72].

#### 4.3.4. Determination of Oxidative Stress Markers

Oxidative stress markers were assessed in tissue homogenates, plasma, and erythrocytes. The concentration of TBARS, as a marker of lipid peroxidation intensity, was measured by reacting TBA with MDA under acidic, high-temperature conditions, with absorbance read at 532 nm using an ELISA reader (Multiskan Ascent No354, Thermo Labsystems, Vantaa, Finland). TBARS concentration in kidney tissue was determined based on a molar extinction coefficient of 1.56 × 105 M^−1^ cm^−1^ and expressed in nmol per mg protein [73]. Erythrocyte TBARS content was measured after resuspending washed erythrocytes in phosphate buffer, adding TCA, and reading the resulting absorbance. The TBARS content was expressed in nanomoles per milliliter of erythrocytes [74].

Catalase (CAT) activity was determined by forming a yellow complex between H_2_O_2_ and molybdenum salts, following Goth’s method [75]. Tissue homogenates or plasma (100 µL) were incubated with H_2_O_2_, and plasma samples received TCA before centrifugation. The absorbance of the supernatant, combined with ammonium-molybdate, was read at 410 nm. CAT activity expressed as mmol per mg of protein for the tissue homogenates and in catalytic units per liter of plasma (U/L) quantifies the enzyme’s ability to decompose 1 μmol of H_2_O_2_ in a unit of time under these conditions, with a standard for comparison.

The concentration of reduced glutathione (GSH) in kidney tissue was assessed using Ellman’s reagent (DTNB) according to Tietze’s method. Tissue homogenates were prepared in Na_2_EDTA buffer, and the absorbance of the colored complex was measured at 412 nm after 3 min, with GSH levels expressed in nmol per mg protein [76].

#### 4.3.5. Determination of Inflammatory Markers

Concentrations of tumor necrosis factor-*α* (TNF-*α*), interleukin-6 (IL-6), and interleukin-1*β* (IL-1*β*) were determined in plasma and supernatants of 2% homogenates of fresh kidney tissue. For determining the concentrations of TNF-*α* and IL-6 in plasma and tissue homogenates, and IL-1*β* in tissue homogenates of rats, commercial ELISA kits (Enzyme-linked immunosorbent assay) specific for rat biological material were used, following the manufacturer’s instructions (R&D Systems^®^ Quantikine^®^ ELISA Kit, Minneapolis, MN, USA). Absorbance was read on an ELISA reader (Multiskan Ascent No354, Thermo Labsystems, Helsinki, Finland) at 450 nm. Concentration values of inflammation parameters in plasma and tissues were calculated from the standard curve, and the results are presented as pg of TNF-*α*, IL-6, and IL-1*β* per mL of plasma or mg of protein in rat kidney tissue.

#### 4.3.6. Histopathological Examination

Kidney tissue from all experimental groups was promptly fixed in a 10% buffered formaldehyde solution (*v*/*v*; pH = 7.4) immediately after isolation and left for 24 h. Tissue sections, 5 mm thick, underwent standard processing at the Center for Pathology and Pathological Anatomy, Faculty of Medicine, University of Niš, using an autotechnikon (Leica Microsystems, Reuil-Malmaison, France). Following the creation of paraffin molds, 4 µm thick sections were obtained using a microtome (Leica Biosystems RM2245, Nussloch, Germany) and subjected to staining using both the standard hematoxylin and eosin (H&E) technique and periodic acid-Schiff staining (PAS).

Semiquantitative analysis of the tissue samples was carried out using an Olympus BKS50 light microscope (Olympus, Tokyo, Japan), equipped with a Leica DFC 295 digital camera (Leica Microsystems, Nussloch, Germany), at various magnifications. H&E staining was employed for the evaluation of morphological changes in kidney tissues, while PAS staining was utilized for the examination of the basement membrane of glomerular capillary loops and tubular basement membrane.

The semiquantitative assessment of kidney tissue damage was performed based on a predefined scale: degree of damage up to 25% (−), from 25 to 50% (+), from 50 to 75% (++), and greater than 75% (+++).

### 4.4. Statistical Analysis

The obtained results were statistically processed using the SPSS software package (version 20.0, SPSS, Inc., Chicago, IL, USA). The results are presented in tabular and graphical form. Continuous variables are shown as mean and standard deviation. The normality of the distribution of continuous variables was determined using the Shapiro-Wilk test. Comparison of variable values between two groups was performed using the Student’s *t*-test for independent samples if the distribution was normal, or the Mann-Whitney test if the distribution deviated from normal. For comparison of variables between multiple groups, analysis of variance (ANOVA) was used if the distribution was normal, or the Kruskal-Wallis test if the distribution deviated from normal. In the case of ANOVA, a corresponding Post Hoc analysis was also performed to compare variables individually between each modality of the same categorical variable. A significance level of *p* < 0.05 was considered statistically significant.

## 5. Conclusions

Taken together, the research findings indicate that chokeberry products and by-products could be considered potential pharmaceuticals for kidney protection against cisplatin-induced toxicity. Treatment with lyophilized chokeberry preparations in cisplatin-treated rats improved oxidative stress and inflammatory markers in kidney tissue and plasma, thereby reducing the side effects of this cytostatic. The results suggest the need for further studies on chokeberry and other polyphenol-rich plants to better understand their therapeutic and preventive effects. This is particularly significant for the utilization of waste products, which have shown substantial protective activity and a high content of biologically active compounds. The research highlights the multifaceted potential of chokeberry as a valuable asset in promoting human health and well-being, both as a functional food and as a candidate for therapeutic and sustainable applications.

## Figures and Tables

**Table 1:** Total phenolics, anthocyanins, flavonoids, proanthocyanidins content, and individual compounds HPLC quantification in LCE, LCWE and LCJ.

	LCE	LCWE	LCJ	*p*
Compounds	Amounts			
Total phenolics content *	328.7 ± 24.23 ^a^	654.82 ± 45.71 ^b^	447.25 ± 52.34 ^c^	0.001
Total anthocyanins **	134.8 ± 24.35 ^a^	467.28 ± 32.72 ^b^	142.54 ± 17.81 ^a^	0.001
Total flavonoids ***	52.51 ± 4.32 ^a^	33.67 ± 3.28 ^a^	32.28 ± 3.52 ^a^	0.000
Total proanthocyanidins ****	102.37 ± 25.2 ^a^	131.62 ± 10.1 ^a^	111.6 ± 5 + 7.02 ^a^	0.034
Chlorogenic acid ^#^	2.87 ± 0.1 ^a^	4.28 ± 0.03 ^b^	3.06 ± 0.09 ^a^	0.027
Cyanidin-3-*O*-galactoside ^#^	1.23 ± 0.02 ^a^	7.04 ± 0.07 ^b^	2.02 ± 0.08 ^a^	0.000
Cyanidin-3-*O*-glucoside ^#^	0.11 ± 0.06 ^a^	0.83 ± 0.01 ^b^	0.16 ± 0.01 ^a^	0.000
Cyanidin-3-*O*-arabinoside ^#^	0.48 ± 0.01 ^a^	3.07 ± 0.03 ^b^	0.54 ± 0.08 ^a^	0.000
Quercetin-3-*O*-rutinoside (rutin) ^#^	0.25 ± 0.02 ^a^	0.05 ± 0.00 ^b^	0.2 ± 0.02 ^c^	0.001
Quercetin-3-*O*-galactoside (hyperoside) ^#^	0.4 ± 0.06 ^a^	Traces	0.01 ± 0.00 ^b^	0.001
Quercetin-3-*O*-glucoside (isoquercitrin) ^#^	0.2 ± 0.01 ^a^	Traces	0.02 ± 0.00 ^b^	0.000

Each value represents the mean of *n* = 3 ± standard deviation. * mg GAE/g of LCE, LCWE or LCJ. ** mg cyanidin-3-*O*-glucoside equivalents/g of LCE, LCWE or LCJ. *** mg catechin/g of LCE, LCWE or LCJ. **** mg catechins equivalent/g of LCE, LCWE or LCJ. # mg/g of LCE, LCWE or LCJ. a,b,c Different letters in the rows indicate a statistically significant difference in the content of active compounds between LCE, LCWE and LCJ, based on the ANOVA test (Tukey post-hoc, *p* < 0.05).

**Table 2:** Morphological alterations in kidney tissue.

Morphological Alterations in Kidney Tissue	GROUP							
	C	LCE	LCWE	LCJ	LCE + CIS	LCWE + CIS	LCJ + CIS	CIS
Desquamation of tubular epithelium	**−**	**−**	**−**	**−**	**−**	**++**	**++**	**+++**
Necrosis of proximal tubules	**−**	**−**	**−**	**−**	**−**	**+**	**+**	**+**
Necrosis of distal tubules	**−**	**−**	**−**	**−**	**−**	**+**	**+**	**+**
Damage to the brush border	**−**	**−**	**−**	**−**	**+**	**++**	**+++**	**+++**
Vacuolar degeneration of tubular epithelium	**−**	**−**	**−**	**−**	**+**	**+++**	**+++**	**+++**
Fatty degeneration	**−**	**−**	**−**	**−**	**+**	**+++**	**+++**	**+++**
Congestion of glomeruli	**−**	**−**	**−**	**−**	**+**	**++**	**++**	**++**
Congestion of intratubular capillaries	**−**	**−**	**−**	**−**	**+**	**++**	**+++**	**+++**
Inflammatory leukocyte infiltrate	**−**	**−**	**−**	**−**	**−**	**++**	**++**	**++**
Reduction in glomerular diameter	**−**	**−**	**−**	**−**	**−**	**+**	**+**	**+**

C—control group of healthy rats; LCE, LCWE, LCJ—control groups of healthy rats receiving lyophilized chokeberry preparartions (extract, waste extract, and juice) orally for 10 days; CIS—effects of cisplatin (8 mg/kg, i.p.); LCE + CIS, LCWE + CIS, LCJ + CIS—protective effects of the chokeberry preparations in cisplatin-induced nephrotoxicity. The degree of damage is up to 25% (−), from 25 to 50% (+), from 50 to 75% (++), and greater than 75% (+++).

## Data Availability

Data are contained within the article.
